# Genetic Polymorphisms and Clinical Features in Diabetic Patients With Fatty Liver: Results From a Single-Center Experience in Southern Italy

**DOI:** 10.3389/fmed.2021.737759

**Published:** 2021-10-21

**Authors:** Rosanna Villani, Grazia Pia Magnati, Giuseppe De Girolamo, Moris Sangineto, Antonino Davide Romano, Tommaso Cassano, Gaetano Serviddio

**Affiliations:** ^1^C.U.R.E. (University Centre for Liver Disease Research and Treatment), Liver Unit, Department of Medical and Surgical Sciences, University of Foggia, Foggia, Italy; ^2^Department of Medical and Surgical Sciences, University of Foggia, Foggia, Italy

**Keywords:** diabetes, NAFLD, SNPs, fatty liver, polymorphisms

## Abstract

Genetic background may be involved in the promotion and progression of non-alcoholic fatty liver disease (NAFLD). Previous studies have suggested that the single nucleotide polymorphisms (SNPs) may be associated with the specific clinical features in the patients with hepatic steatosis; however, data on the patients with diabetes from Southern Italy are lacking. We enrolled 454 patients and 260 of them had type 2 diabetes. We studied the *PNPLA3 rs738409, LPIN1 rs13412852, KLF6 rs3750861, SOD2 rs4880, TM6SF2 rs58542926*, and *ZNF624 rs12603226* SNPs and their distribution in the study population. Lipid profile, liver stiffness, and kidney function were also studied to understand the potential role of the SNPs in the development of clinical phenotypes. No differences were observed in the distribution of polymorphisms between the diabetic and non-diabetic subjects. Carriers of risk allele G for *PNPLA3 rs738409* SNP showed a lower mean value of serum triglycerides and a higher liver stiffness. Risk allele for *KLF6 rs3750861* and *SOD2 rs4880* polymorphism had a lower estimated glomerular filtration rate (eGFR) value, whereas no differences in the glucose and glycated hemoglobin level were observed in the subgroups by the different genotypes. Genetic polymorphisms are useful to identify the patients at higher risk of development of liver fibrosis and lower eGFR values in the patients with diabetes and NAFLD. Their use in clinical practice may help the clinicians to identify the patients who require a more strict follow-up program.

## Introduction

Non-alcoholic fatty liver disease (NAFLD) is a major cause of chronic liver disease and the second leading cause of liver transplantation (1). NAFLD has been predominantly associated with diabetes and obesity, and its worldwide prevalence has been estimated to be up to 33% in the general population and 75% in diabetic patients (2). From a clinical point of view, NAFLD encompasses a spectrum of diseases ranging from simple steatosis without liver inflammation to steatohepatitis and, finally, liver cirrhosis (3).

There is a significant heterogeneity in the clinical phenotype and natural history of NAFLD because it is a multifactorial disease resulting from the interaction between the genetic background and environmental factors (4). Obesity, type 2 diabetes, reduced physical activity, and genetic variants are the most important risk factors; however, it is unclear which of them plays a decisive role in the disease promotion and progression. Therefore, in clinical practice, it is unknown which patients have the greatest risk of suffering from liver damage or developing liver cirrhosis (4).

The genetic contribution to NAFLD development has been previously studied and up to now the genome-wide association studies (GWASs) have greatly contributed to understanding the role of the genetic factors in NAFLD pathogenesis and variability of prognosis (5). For example, the single nucleotide polymorphisms (SNPs) in phospholipase domain-containing 3 (*PNPLA3*) and transmembrane 6 superfamily member 2 (*TM6SF2*) gene have been recently associated with the development of steatosis, NAFLD-related hepatocellular carcinoma (HCC), and the stage of liver fibrosis (6–8). On the other hand, type 2 diabetes is considered *per se* an independent risk factor for liver disease progression (9); however, it is not known whether differences in the genetic background of the diabetic population may account for promoting metabolic liver disease or have a minor role because metabolic profile plays a key role in the disease progression. Only few studies have addressed this topic and, therefore, data are currently limited and inconclusive.

Kogiso et al. studied 272 Japanese patients with diabetes and found that the frequency of the risk allele G of *PNPLA3* gene was not different between the diabetic and non-diabetic subjects even if the diabetic patients with the GG genotype have a lower reduction in hemoglobin A1c (HbA1c) level after starting the antidiabetic treatment (10). Luukkonen et al. studied a large population of the obese individuals from Finland carrying the *PNPLA3 I148M* variant and found that the GG genotype was associated with an antiatherogenic lipid profile, decreased very-low-density lipoprotein (VLDL) and low-density lipoprotein (LDL) particles, but increased liver fat content due to a loss-of-function mutation leading to impaired hepatocellular triglyceride hydrolysis (11). Rüschenbaum et al. confirmed these results in a German population; however, only a small number of the patients with diabetes were included in the study and the results were not definitive (12).

It much still needs to be done to understand the potential role of specific genetic background on the development of metabolic liver disease because there are several confounding factors.

This study presents the results of our experience with an Italian cohort of the diabetic patients from Southern Italy and discusses the potential utility of the polymorphisms in clinical practice.

## Methods

### Study Population

We conducted a retrospective cohort study using data from 454 patients who were referred to the Ultrasound Clinic of the University Centre for Liver Disease Research and Treatment of the University of Foggia between March 2016 and March 2020. The study population included patients with the type 2 diabetes (*N* = 260) and non-diabetic individuals (*N* = 194).

At the recruitment, anthropometric, clinical, and biochemical data were recorded.

Moreover, all the patients underwent medical history, abdominal B-mode ultrasound (US), and transient elastography at baseline.

Ultrasound examination was performed by using EPIQ7 US system (Philips Medical Systems International, Best, Netherlands) and a C5-C1 convex probe. The diagnosis of fatty liver was based on the brightness of the liver tissue on US compared with the kidney, vascular blurring of the hepatic vein trunk, and deep attenuation in the right hepatic lobe. The severity of fatty liver change was classified as absent; mild fatty liver in case of a mild increase in hepatic echogenicity with normal visualization of the portal vessels and diaphragm; moderate fatty liver in case of moderate hepatic echogenicity, reduced visualization of the portal vessels, and diaphragm; and, finally, severe fatty liver when a marked increase in echoes was detected in the parenchyma with poor or no visualization of diaphragm.

The liver stiffness evaluation was performed by FibroScan (Echosens, Paris, France) and expressed as KPa. The criteria for a valid examination were at least 10 valid measurements, a ratio of the valid measurements to the total number of the valid and invalid measurements (success rate) > 60% and interquartile range (IQR) < 30% of the median value. Liver stiffness ≥ 7.8 KPa was used as a cutoff value for F2 stage of liver fibrosis and considered as clinically significant (13).

Patients with liver stiffness ≥ 10.5 KPa underwent liver biopsy to avoid misclassification of fibrosis stage (13, 14).

The criteria for the definition of liver cirrhosis were: (a) biopsy-proven presence of the regenerative nodules and fibrous tissue (histological criteria) and (b) presence of the clinical signs of decompensated disease (ascites, jaundice, and variceal bleeding) and/or portal hypertension (splenomegaly and esophageal varices) (clinical criteria).

Inclusion criteria were:

- Diagnosis of NAFLD ± Type 2 diabetes

Non-alcoholic fatty liver disease was defined by the excessive hepatic fat accumulation detected by ultrasonography or liver biopsy in the absence of the secondary causes of fatty liver.

Secondary causes of fatty liver were:

- Alcohol use disorders defined according to the European Association for the Study of the Liver (EASL) guidelines (15)
- Hepatitis C virus-associated fatty liver
- Autoimmune hepatitis
- Inherited liver disorders (hemochromatosis, Wilson disease, celiac disease, and abeta-/hypobetalipoproteinemia)
- Drug-induced liver injury

Patients aged < 18 years with type 1 diabetes mellitus (T1DM), hepatitis B virus (HBV), or HIV infection were also excluded from the final analysis.

Patients with diabetes received one of the following antidiabetic regimens:

(a) Non-insulin therapy (use of the oral antidiabetic agents)

(b) Basal regimen (use of the basal insulin ± oral antidiabetic agents)

(c) Basal-bolus regimen (combination of basal insulin with a rapid-acting insulin at mealtimes).

Glycated hemoglobin was assessed from 8 weeks to 12 weeks after changing the antidiabetic medications.

Dyslipidemia was defined as the serum triacylglycerols > 150 mg/dl or total cholesterol level ≥ 200 mg/dl or LDL level ≥ 150 mg/dl or lipid-lowering treatment.

All the patients provided a signed informed consent and the study protocol was conducted according to the principles reported in the Declaration of Helsinki. The protocol was approved by the local Institutional Review Board. All the data generated or analyzed during this study are included in this published article.

### Genetic Analysis

The *PNPLA3 rs738409* C→ G, *LPIN1 rs13412852* T→ C, *KLF6 rs3750861* T→ C, *SOD2 rs4880* C→ T, *TM6SF2 rs58542926* C→ T, and *ZNF624 rs12603226* C→ T SNPs were genotyped.

Genomic DNA was extracted from whole blood using the MagCore® Nucleic Acid Extractor, Bioscience, Taiwan.

The polymorphism analysis was done by real-time polymerase chain reaction (rt-PCR) by using a commercial kit (FLT PLUS Fatty Liver Test; Orga Bio Human, Rome, Italy) as instructed by the manufacturer.

The distribution of the allelic frequencies was studied in the overall population and the subgroups to show the potential deviations from the Hardy–Weinberg equilibrium (HWE).

### Statistical Analysis

Statistical analysis was performed by using the SPSS (Statistical Package for the Social Sciences, version 20, Armonk, New York, USA) and GraphPad Prism version 8 (GraphPad Software, La Jolla, California, United States of America). Categorial data were reported as the absolute numbers (percentages) and the continuous variables as mean ± SD. The comparisons between the groups were performed by using ANOVA, Kruskal–Wallis test, chi-squared test, or Fisher's exact test where appropriate. Two-tailed *p* < 0.05 were considered as statistically significant. Sample size was not calculated due to the exploratory design of the study.

## Results

### Study Population

Baseline characteristics of the study population are reported in Table 1. There was a statistically significant difference between the subgroups by age; therefore, age-adjusted results are shown below.

**Table 1:** Baseline characteristics of study population.

	**All**	**DM+**	**DM–**	***p***
	**(*N* = 454)**	**(*N* = 260)**	**(*N* = 194)**	
Age (years)	58.5 (13.6)	63.8 (9.7)	58.6 (13.4)	<0.001
Sex (M/F)-*N* (%)	244 (53.7%)/210 (46.3%)	142 (54.6%)/118 (45.4%)	102 (52.6%)/92 (47.4%)	0.66
BMI (Kg/m^2^)	29.4 (5.6)	30.5 (5.5)	27.6 (5.2)	<0.001
Waist circumference (cm)	101.2 (14.3)	105.5 (13)	93.3 (12.3)	<0.001
NAFLD-*N* (%)	301 (62.3%)	205 (78.8%)	96 (49.5%)	<0.001
Cirrhosis-*N* (%)	23 (5%)	23 (8.8%)	–	<0.001
Dyslipidemia-*N* (%)	243 (53.5%)	197 (75.8%)	46 (23.7%)	<0.001

*Age, BMI, and waist circumference are expressed as mean ± SD*.

*BMI, body mass index; NAFLD, non-alcoholic fatty liver disease*.

The prevalence of NAFLD was significantly higher in the diabetic population than in non-diabetic group (78.8 vs. 49.5%; *p* < 0.001). Twenty-three patients with diabetes and NAFLD had liver cirrhosis, whereas no patients had advanced liver fibrosis in the control group. As expected, there was a notably higher prevalence of dyslipidemia in the diabetic patients than in control group (75.8 vs. 23.7%; *p* < 0.001). In subgroup analysis by the antidiabetic treatment, the patients treated with non-insulin therapy had significantly lower HbA1c levels (mean 6.9 ± 1.36%) compared with the patients treated with basal regimen (mean 8.06 ± 1.44%), whereas the patients treated with basal-bolus therapy had a mean HbA1c of 8.37 ± 1.08% (non-insulin therapy group vs. basal regimen group *p* < 0.001; non-insulin therapy group vs. basal-bolus regimen group *p* < 0.001; and basal regimen group vs. basal-bolus regimen group *p* = 0.16).

### Frequency of SNPs in the Study Population

**Figure 1:**
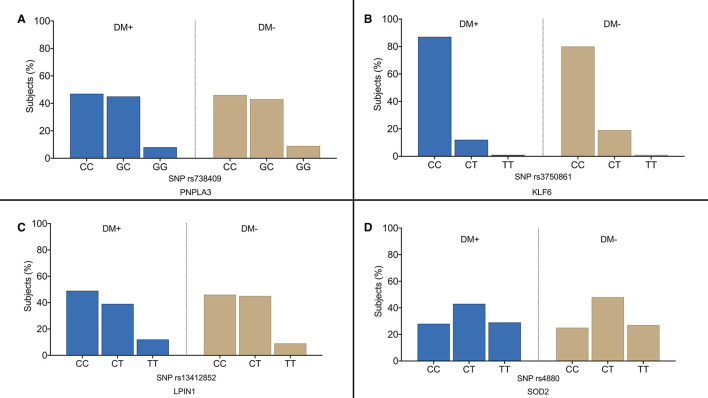
Overall distribution of *PNPLA3 rs738409* **(A)**, *KLF6 rs3750861* **(B)**, *LPIN1 rs13412852* **(C)**, and *SOD2 rs4880* **(D)** single nucleotide polymorphisms (SNPs) in our study population. DM+, patients with type 2 diabetes; DM−, non-diabetic individualsof.

Figure 1 shows the overall distribution of *PNPLA3 rs738409* (Figure 1A), *KLF6 rs3750861* (Figure 1B), *LPIN1 rs13412852* (Figure 1C), and *SOD2 rs4880* SNPs (Figure 1D). No differences were observed in the distribution of these polymorphisms between the diabetic and non-diabetic patients. Similarly, *TM6SF2 rs58542926* and *ZNF624 rs12603226* SNPs did not show the different distribution between the two groups (data not shown). Table 2 shows the genotype distribution that was in HWE. The risk allele frequency, which is also reported in Table 2, was not statistically different between the diabetic and non-diabetic patients.

**Table 2:** Frequency of *PNPLA3 rs738409, LPIN1 rs13412852, SOD2 rs4880, KLF6 rs3750861*, and *TM6SF2 rs58542926* risk alleles in the study population.

	**HWE *p***	**Minor allele**	**Risk allele**	**Risk allele frequency overall**	**Risk allele frequency diabetic patients**	**Risk allele frequency diabetic patients with NAFLD**	**Risk allele frequency Non-diabetic patients**
PNPLA3 rs738409	0.968	G	G	0.311	0.308	0.343	0.317
LPIN1 rs 13445678	0.850	T	C	0.665	0.687	0.673	0.678
SOD2 rs 4880	0.486	T	T	0.504	0.503	0.517	0.505
KLF6 rs 3750861	0.992	T	C	0.918	0.934	0.923	0.895
TM6SF2 rs 58542926	0.998	T	C	0.958	0.973	0.968	0.935

*HWE, Hardy–Weinberg equilibrium; NAFLD, non-alcoholic fatty liver disease*.

### Single Nucleotide Polymorphisms and Prevalence of NAFLD in the Diabetic Population

**Figure 2:**
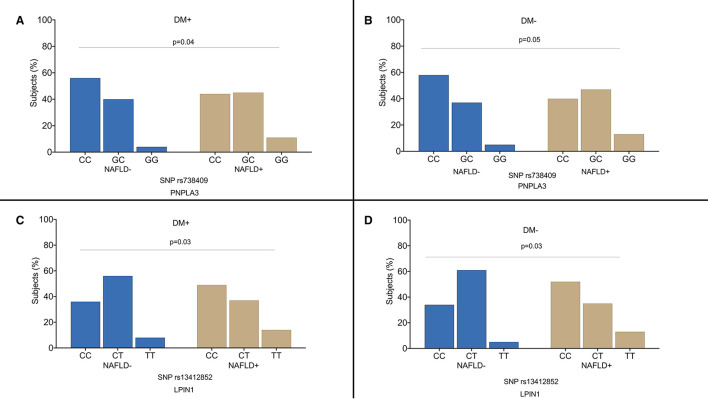
Distribution of the genotypes in the diabetic patients **(A–C)** and non-diabetic individuals **(B–D)** according to the presence of non-alcoholic fatty liver disease (NAFLD). DM+: patients with type 2 diabetes; DM−: non-diabetic individuals.

Figure 2 shows the distribution of genotypes in the diabetic and non-diabetic patients according to the presence of NAFLD. Both in the diabetic and non-diabetic patients, we observed a higher frequency of risk allele for *PNPLA3 rs738409* (*p* = 0.04) (Figures 2A,B) and *LPIN1 rs13412852* (*p* = 0.05) SNPs (Figures 2C,D). Particularly, both the patients with diabetes and non-diabetic ones had a higher frequency of *PNPLA3 rs738409*G allele in NAFLD group (GG genotype prevalence was 11% in the NAFLD patients vs. 4% in control group and GC genotype prevalence was 45% in NAFLD group vs. 40% in the individuals without liver steatosis). The mean age of the patients was not different in the subgroups by *PNPLA3 rs738409* SNPs (the diabetic patients CC genotype 62.7 ± 11.2 years vs. CG genotype 64.8 ± 8.6 years vs. GG genotype 63.6 ± 9.3 years and the non-diabetic patients CC genotype 58.4 ± 10.2 years vs. CG genotype 57.8 ± 12.3 years vs. GG genotype 59.3 ± 13.9 years).

Regarding *LPIN1 rs13412852* SNP distribution, the risk allele C was more frequently observed in the patients with hepatic steatosis irrespective of the history of diabetes. The frequency of the different genotypes was similar in the diabetic vs. control group for *KLF6 rs3750861, SOD2 rs4880, TM6SF2 rs58542926*, and *ZNF624 rs12603226* SNPs.

### Single Nucleotide Polymorphisms and Lipid Profile

We analyzed the lipid profile of the diabetic patients according to the genotypes. In the subgroup analysis by *PNPLA3 rs738409* genetic variants, no differences were observed between the different genotypes in the serum total cholesterol and HDL levels, whereas the patients with the risk allele G showed a lower mean value of serum triglycerides (172.5 mg/dl for CC genotype, 121 mg/dl for CG genotype, and 117 mg/dl for GG genotype; CC vs. CG *p* < 0.001; CC vs. GG *p* < 0.001). Table 3 shows no differences that were found in total cholesterol, HDL, and triglycerides serum levels in the patients according to *LPIN1 rs13412852, KLF6 rs3750861*, and *SOD2 rs4880 SNPs*. Similarly, no differences in lipid profile were observed in the diabetic patients according to *TM6SF2 rs58542926* and *ZNF624 rs12603226* SNPs (data not shown).

**Table 3:** Lipid profile in the diabetic patients according to the SNPs and genotypes.

**SNP**	**Genotype**	**Total cholesterol**	**LDL cholesterol**	**HDL cholesterol**	**Triglycerides**
		**(mg/dl)**	**(mg/dl)**	**(mg/dl)**	**(mg/dl)**
PNPLA3 rs738409	C/C	178.9, 43.1	112.2, 35.5	46.8, 11.4	172.5, 68.8
	C/G	178.5, 43.8	115.6, 33.3	47.7, 17.9	121, 73.1^*^
	G/G	162.7, 46.7	110.8, 28.6	43.8, 9.1	117.4, 63.1^§^
LPIN1 rs13445678	C/C	183.7, 52.5	118.4, 31.6	49.5, 16.7	166.3, 68.8
	C/T	168.9, 45.1	117.1, 34.3	46.1, 12.9	146.2, 78.7
	T/T	169.2, 33.9	169.2, 33.9	45, 11.1	162.1, 97.4
SOD2 rs4880	C/C	186.2, 44.5	118.1, 36.7	48.6, 12.9	167.6, 90.7
	C/T	179.4, 48.8	108.9, 27.6	46.6, 17	166.3, 110.3
	T/T	176.9, 46.7	113.2, 35.2	48.3, 13.3	157.9, 69.4
KLF6 rs3750861	C/C	176.2, 42.7	111.2, 31.9	47.3, 12.1	154.2, 92.8
	C/T	184.3, 51.2	122.5, 36.3	48.7, 27	179.1, 114.9

*SNPs, single nucleotide polymorphisms; LDL, low-density lipoprotein; HDL, high-density lipoprotein*.

* *p < 0.001 for comparison genotype CC vs CG*.

§ *p < 0.001 for comparison genotype CC vs. GG*.

### Single Nucleotide Polymorphisms and Kidney Function

**Figure 3:**
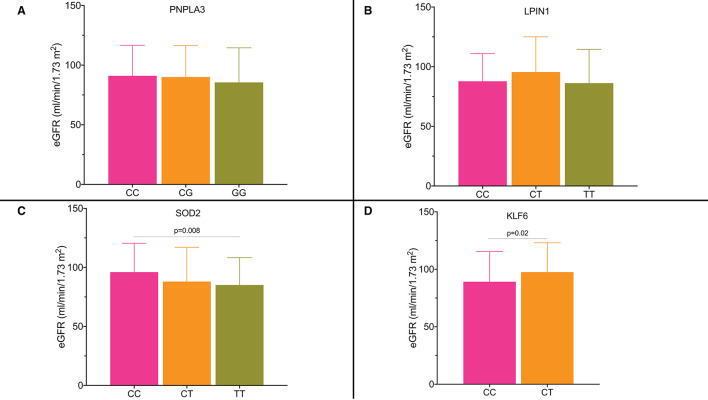
**(A–D)** Estimated glomerular filtration rate (eGFR) according to the single nucleotide polymorphisms and their genotypes.

We analyzed the serum creatinine and estimated glomerular filtration rate (eGFR) values in the subgroups by the genotypes. Figures 3A,B shows that there was no association between kidney function and *PNPLA3 rs738409* and *LPIN1 rs13412852* SNPs. On the other hand, there was a statistically significant reduction in eGFR value for *SOD2 rs4880* (Figure 3C) and *KLF6 rs3750861* SNPs (Figure 3D). Particularly, the patients who carried the risk allele T for *SOD2 rs4880* SNP showed a reduction in eGFR value (CC genotype 96.2 ± 24.4 ml/min/1.73 m^2^; CT genotype 88.3 ± 29 ml/min/1.73 m^2^; TT genotype 85.1 ± 23.2 ml/min/1.73 m^2^; CC vs. TT genotype *p* = 0.008). Patients who carried allele T for *SOD2 rs4880* SNP had an odds ratio (OR) of 1.62 (95% CI: 1.06–2.56) for eGFR value <90 ml/min/1.73 m^2^.

The risk allele C for *KLF6 rs3750861* polymorphism was also associated with a significant reduction in eGFR value [CT genotype 97.5 ± 25.6 ml/min/1.73 m^2^ vs. TT genotype 89.2 ± 26.3 ml/min/1.73 m^2^ (*p* = 0.02)]. Serum creatinine values were not different in the subgroups by the genotypes for all the available SNPs. The OR for eGFR value <90 ml/min/1.73 m^2^ in the individuals who carried allele C was 1.95 (95% CI: 1.21–3.15).

### Single Nucleotide Polymorphisms and Liver Fibrosis

**Figure 4:**
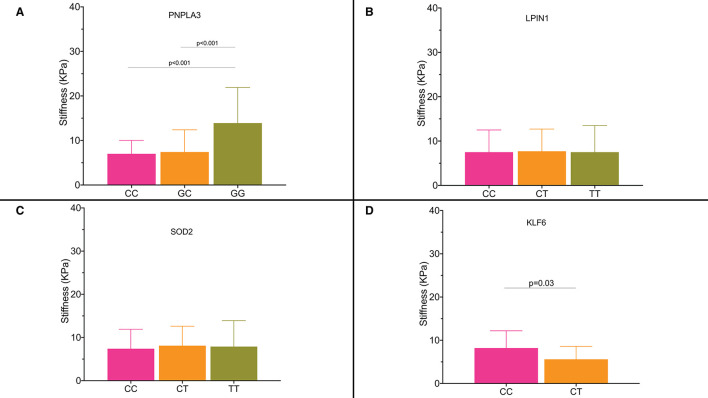
**(A–D)** Liver stiffness expressed as KPa according to the single nucleotide polymorphisms and their genotypes.

Figure 4 shows liver stiffness values for the diabetic patients according to genotype. Genotype GG for *PNPLA3 rs738409* SNP was associated with significantly higher liver stiffness values compared with the CC and CG genotypes (GG: 13.9 ± 8.2 KPa; CG: 7.4 ± 5.1 KPa; CC: 7 ± 3.4 KPa; CC vs. CG genotype *p* < 0.001; CC vs. GG genotype *p* < 0.001) (Figure 4A). Patients who carried the *PNPLA3 rs738409* allele had a GWAS that was associated with an age-adjusted OR for significant fibrosis of 1.44 (95% CI: 1.03–2.26).

Similar results were observed for the patients with *KLF6 rs3750861* CC genotype who had a higher liver stiffness (8.2 ± 4 KPa) compared with the patients with CT genotype (5.6 ± 3.3 KPa; *p* = 0.03) (Figure 4D).

Patients who carried *KLF6 rs3750861* allele C had an OR of 1.98 (95% CI: 1.13–5.51) for significant fibrosis.

Our cohort of the patients included 23 patients with the diabetes and biopsy-proven metabolic-related cirrhosis. We analyzed their genotypes and found that all the patients except for four had GG genotype for *PNPLA3 rs738409* SNP, whereas all the patients except for one had at least one allele risk for *LPIN1 rs13412852* SNP. All the patients with cirrhosis had at least one risk allele for *SOD2 rs4880* SNP too. Patients included in the non-diabetic group did not show different stiffness values by the genotypes for all the studied SNPs (data not shown).

### Single Nucleotide Polymorphisms and Glucose Control

Glucose level and HbA1c were analyzed in the subgroups by the SNPs and, for each polymorphism, data were analyzed by antidiabetic therapy (non-insulin treatment vs. insulin therapy groups). The mean values of glycated hemoglobin in the patients by *PNPLA3 rs738409* SNP genotype were not statistically different even if a trend toward increased levels was observed in the patients with the GG genotype both in the patients treated with insulin and in non-insulin-treated subjects. Similarly, no significant results were observed for the HbA1c levels by *LPIN1 rs13412852, KLF6 rs3750861, SOD2 rs4880, TM6SF2 rs58542926*, and *ZNF624 rs12603226* SNPs. No differences in the glucose levels were observed in all the subgroups by the SNPs and antidiabetic treatment.

## Discussion

Non-alcoholic fatty liver disease affects almost two billion people globally and its burden is expected to grow in the coming decades. The prevalence estimates indicate that it is the most prevalent liver disease in human history (16). NAFLD is becoming an established risk factor for the cardiovascular disease and type 2 diabetes, which are currently the leading causes of death and disability (16).

The identification of familiar clustering and interethnic differences in susceptibility has suggested that a significant heritable component may be involved in the development and progression of NAFLD (17, 18). The availability of the GWASs in large cohorts of the patients with NAFLD has enabled the researchers to identify the potential genetic factors that could predispose the individuals to the clinically relevant consequences such as liver cirrhosis (17). However, although several studies have improved our understanding of the role of genetic background on NAFLD pathogenesis and the potential mechanisms underlying fat accumulation and fibrosis development, at present, the predictive power of the genetic factors is still too limited to support their daily use in clinical practice.

A large number of studies have found that the several gene variants for *PNPLA3, TM6SF2*, glucokinase regulatory protein (*GCKR*), lysophospholipase-like 1 (*LYPLAL1*), or membrane-bound O-acyltransferase domain-containing 7 (*MBOAT7*) significantly increase the risk of NAFLD (19). Some of them are associated with an increase in the risk of diabetes and liver disease and others are involved only in NAFLD development. For example, *PNPLA3 rs738409* GG genotype carriers have a higher risk of developing fatty liver (73%) than non-carriers (20%) but a small increase in the risk of type 2 diabetes (21), whereas a *TM6SF2* gene variant is associated with a 2.1-fold increase in the risk of NAFLD and a 40% increase in the incidence of diabetes (19, 22). Most available data have been obtained from the international studies and, currently, few studies are available concerning the Italian population.

Bellan et al. studied the impact of *PNPLA3 rs738409* SNPs in the prediction of hepatic steatosis and fibrosis in a cohort of 328 patients from the Northern Italy and found that the carriage of the G allele was associated with higher liver stiffness values (5.9 kPa in CC homozygotes, 6.1 kPa in CG heterozygotes, and 6.8 kPa in GG homozygotes; *p* = 0.01), whereas no differences were found in glycated hemoglobin in the subgroups by the genotypes (23). The impact of *PNPLA3* polymorphisms on the onset and severity of liver disease in the subgroup of the patients with diabetes was also investigated; however, the strength of this association was irrelevant and the authors concluded that confounding factors, such as body mass index (BMI), could play a major role in the development of liver disease in the diabetic population (23).

Grimaudo et al. studied the impact of genetic background on the development of liver disease in a large population from the Southern Italy; however, only 46% had diabetes and the study population included only the patients with NAFLD-related cirrhosis (24).

In this study, we found that the genotype distribution of *PNPLA3 rs738409, TM6SF2 rs58542926, LPIN1 rs13412852, KLF6 rs3750861, SOD2 rs4880*, and *ZNF624 rs12603226* SNPs was not different in the diabetic vs. non-diabetic population, whereas their distribution was different between the patients with NAFLD and the patients without NAFLD irrespective of presence of diabetes. The SNPs seemed not to be involved in the pathogenesis of diabetes but rather involved in the hepatic changes regardless of glucose levels.

Our analysis of lipid profile in the diabetic patients by the SNPs did not show any changes in the total cholesterol and LDL cholesterol levels, whereas the serum triglyceride levels were significantly lower in *PNPLA3 rs738409* G allele carriers. *PNPLA3*, also known as adiponutrin, is a member of the patatin-like phospholipase family and *PNPLA3* gene encodes a membrane-bound triacylglycerol lipase that mediates triacylglycerol hydrolysis and its polymorphisms have been widely associated with the progression of liver fibrosis and development of HCC (20, 25–28). The isoleucine-to-methionine substitution at residue 148 variant of *PNPLA3* gene (allele G) is due to a loss-of-function mutation, which leads to impaired hepatocellular triglyceride hydrolysis and, finally, triglyceride accumulation in the hepatocytes (11). Our findings are in accordance with the results reported by Palmer et al. who observed in two large populations including obese patients, paradoxical lower serum triglyceride levels in *PNPLA3 rs738409* G allele carriers in accordance with the impaired hepatic secretion of the triglycerides.

In this study population, we observed the higher stiffness values in the patients with diabetes who carried the GG genotype. These results confirmed the role of *PNPLA3 rs738409* SNPs in the prediction of liver fibrosis in a population of the patients with diabetes from the Southern Italy and suggested that its use in routine clinical practice improves the identification of the patients who are at a higher risk of fibrosis development and progression. Moreover, concordantly with the previously reported data (29, 30), we observed a significant increase in liver stiffness values in the patients with the CC genotype for *KLF6 rs3750861* SNP. *KLF6* is a ubiquitously expressed transcription factor, which is involved in the cell proliferation, differentiation, and cell death (31). It is early expressed in activated hepatic stellate cells (HSCs) after liver injury, suggesting a potential role in the process of liver fibrogenesis (32). Vespasiani-Gentilucci et al. reported a prevalence of CC, CT, and TT genotypes, which are very similar to our population; however, this study population included 290 patients and prevalence of diabetes was only 40%. In this study, the authors did not find a significant difference in the stage of liver fibrosis between CC and TT subgroups (33).

The impact of the SNPs on glucose metabolism of the patients with NAFLD is also a hot topic in hepatology because evidence is inconsistent. Machado et al. reported an unexpected better fasting plasma glucose control in the patients with *PNPLA3 rs738409* GG genotype (34), whereas Petit et al. did not report any differences in fasting plasma glucose and the HbA1c levels by *PNPLA3 rs738409* SNPs (35).

As suggested by Mantovani et al. (36), given that the risk allele G of *PNPLA3 rs738409* is linked to an increased risk of the fibrosis development and progression and that the severity of NAFLD is associated with a worse glycemic control (37, 38), we should not expect the lower glucose and HbA1c levels in the patients who carry the risk allele G of *PNPLA3 rs738409*.

Concordantly, we found no differences in the fasting plasma glucose and HbA1c levels in the patients with the GG genotype vs. CG or CC genotype, showing that our data are consistent with the results previously reported by Mantovani et al. (37). Concerning the potential role of *LPIN1 rs13412852, KLF6 rs3750861, SOD2 rs4880, TM6SF2 rs58542926*, and *ZNF624 rs12603226* SNPs in glycemic control of the patients with diabetes, data are also limited. *LPIN1* is a magnesium-dependent phosphatase responsible for catalyzing the penultimate step in triacylglycerol synthesis and, in addition, it is a transcriptional coactivator that interacts with the nuclear receptor peroxisome proliferator-activated receptor-α (PPARα) and PPARγ coactivator 1α (PPARGC1A) to regulate fatty acid oxidation gene expression (39). Body fat accumulation is a major regulator of *LPIN1* gene expression and this is strongly associated with insulin-mediated subcutaneous adipocyte glucose transport (40). Some authors have investigated a potential role of *LPIN1* SNPs in the phenotype of the patients with metabolic alteration; however, a large number of SNPs have been studied and all of them have confirmed that there are no differences in fasting plasma glucose by *LPIN1* SNPs (39, 41). These results are consistent with our findings. Data on the impact of *KLF6* mutation on the fasting glucose control and insulin level are available for *KLF6 rs3750861* G>A polymorphism, whereas data concerning the potential role of *KLF6 rs3750861* T>C polymorphism on glucose control are very limited (29, 42). Hepatocyte expression of *KLF6* regulates hepatic fatty acid and glucose metabolism via transcriptional activation of liver glucokinase and posttranscriptional regulation of PPARα (43). *KLF6 rs3750861* polymorphisms are associated with the novel binding sites and promotion of alternative splicing of *KLF6* into the truncated isoforms (42). We reported for the first time in a population of the patients with diabetes that *KLF6 rs3750861* T>C polymorphism is not associated with significant differences in fasting plasma glucose and HbA1c levels; however, because of the limited sample size, further confirmation is needed. We found a similar result for *TM6SF2 rs58542926* SNP, which has been previously linked to an increased hazard of developing diabetes (22) and that, on the contrary, did not show a significant impact on glucose control in our study population.

We analyzed the association between the SNPs and kidney function. Some authors have recently suggested a significant association between the SNPs and risk of chronic kidney disease (CKD), but the mechanisms supporting this association are poorly understood. Mantovani et al. have observed in a cohort of diabetic patients (*N* = 112) that the patients homozygous for *PNPLA3 rs738409 I148M* variant (GG genotype) had lower eGFR values and a higher prevalence of CKD independently from liver disease (44). These results were confirmed by the same authors in a group of 101 women with diabetes (37). Sun et al. showed that the patients with NAFLD, normal alanine aminotransferase (ALT) levels, and carriage of *PNPLA3 rs738409* G allele were at higher risk of early glomerular and tubular damage (45).

In our cohort, we found a trend toward the lower eGFR values in the patients with the GG genotype for *PNPLA3 rs738409* polymorphisms without reaching statistical significance. This result could be affected by the small number of the patients included in the GG genotype group. Conversely, we found a significant difference in mean eGFR value for the diabetic patients by *SOD2* and *KLF6* genetic variants.

Several authors have studied the impact of polymorphism T>C of *SOD2 rs4880* on kidney function in the diabetic patients (46). The gene is coded in the nuclear DNA, therefore the enzyme translocates to the mitochondria after translation of the protein in the cytosol. The valine-to-alanine substitution induces the conformational changes with a less efficient transport of *SOD2* into the mitochondrial matrix and, finally, lowers the ability to neutralize the superoxide radicals, which are largely produced in the mitochondria of the patients with hyperglycemia (47).

Mollsten et al. found a significant association between *SOD2* polymorphisms and diabetic nephropathy in the patients with type 1 diabetes (46).

Nomiyama et al. observed a strong association between the CT or CC genotype and risk of nephropathy in a large population (*N* = 478) of the Japanese diabetic patients. Data on the correlation between *SOD2* genotype and risk of CKD are not available for the Italian population and we confirmed for the first time these results in the Italian patients.

Limitations of our study are the sample size, the use of ultrasonography for the detection of fatty liver, and the lack of follow-up data. Long-term follow-up data are needed to understand whether the SNPs can be associated with pivotal clinical outcomes such as the rate of disease progression, the risk of HCC, or the response to a specific class of the antidiabetic drugs.

These clinical issues should be investigated in the future studies to reach one of the most important challenges of the modern medicine, which is the tailoring of medical treatment to the individual characteristics of each patient.

In conclusion, our findings suggest that in diabetic population, the GG genotype of *PNPLA3 rs738409* and the CC genotype of *KLF6 rs3750861* SNPs are associated with the higher stiffness values and the risk of developing liver fibrosis, whereas the CC genotype of *SOD2 rs4880* and the CT genotype of *KLF6 rs3750861* SNPs are associated with the lower eGFR values. Their use in clinical practice may help the clinicians to select the patients with diabetes who require a strict follow-up program.

## Data Availability Statement

The original contributions presented in the study are included in the article/supplementary material, further inquiries can be directed to the corresponding authors.

## Ethics Statement

The studies involving human participants were reviewed and approved by Institutional review Board (University of Foggia). The patients/participants provided their written informed consent to participate in this study.

## Author Contributions

RV and GS contribute to the concept and design of study. GPM, GDG, MS, and ADR contribute to the acquisition of data. RV and GS contribute to the statistical analysis. RV, TC, and GS contribute to the analysis and interpretation of data and contribute to the drafting of the manuscript. RV, GS, GPM, GDG, MS, and ADR contribute to the critical revision of the manuscript. All authors contributed to the article and approved the submitted version.

## Conflict of Interest

The authors declare that the research was conducted in the absence of any commercial or financial relationships that could be construed as potential conflicts of interest.

## Publisher's Note

All claims expressed in this article are solely those of the authors and do not necessarily represent those of their affiliated organizations, or those of the publisher, the editors and the reviewers. Any product that may be evaluated in this article, or claim that may be made by its manufacturer, is not guaranteed or endorsed by the publisher.
